# Supplementary material for: Progesterone Receptor Membrane Component 1 (PGRMC1) Modulates Tumour Progression, the Immune Microenvironment and the Response to Therapy in Glioblastoma
Source: Cells. 2023 Oct 20;12(20):2498. doi: 10.3390/cells12202498 (PMC10604944; doi:10.3390/cells12202498)
Supplement: Supplementary file 1 [file cells-12-02498-s001.zip › cells-2568200-supplementary.pdf]

	Hannover cohort		Magdeburg cohort	
	**number**	**percentage**	**number**	**percentage**
**All patients**	135	100	170	100
**Sex**				
female	52	38.5	78	45.9
male	83	61.5	92	54.1
**KPS**				
10	1	0.7	3	1.8
20	1	0.7	2	1.2
30	1	0.7	10	5.9
40	5	3.7	8	4.7
50	15	11.1	27	15.9
60	27	20	31	18.2
70	25	18.5	54	31.8
80	33	24.4	16	9.4
90	19	14.1	18	10.6
100	1	0.7	0	0
n.d.	7	5.2	1	0.6
**Therapy**				
surgery	15	11.1	34	20
surgery+RTX	21	15.6	17	10
surgery+CTX	3	2.2	0	0
surgery+RTCX	93	68.9	114	67.1
n.d.	3	2.2	5	2.9
**Surgical resection**				
total	52	38.5	59	34.7
subtotal/biopsy	71	52.6	102	60
n.d.	12	8.9	9	5.3
**MGMT methylation**				
unmethylated	62	45.9	62	36.5
methylated	9	43.7	108	63.5
n.d.	14	10.4	0	0
**IDH mutation**				
wild-type	135	100	170	100
mutated	0	0	0	0
n.d.	0	0	0	0

Supplementary Table S1. Clinical characteristics of the GBM patients. KPS: Karnofsky Performance Scale; RTX: radiotherapy; CTX: chemotherapy; RCTX: radio-chemotherapy; n.d.: not determinable.

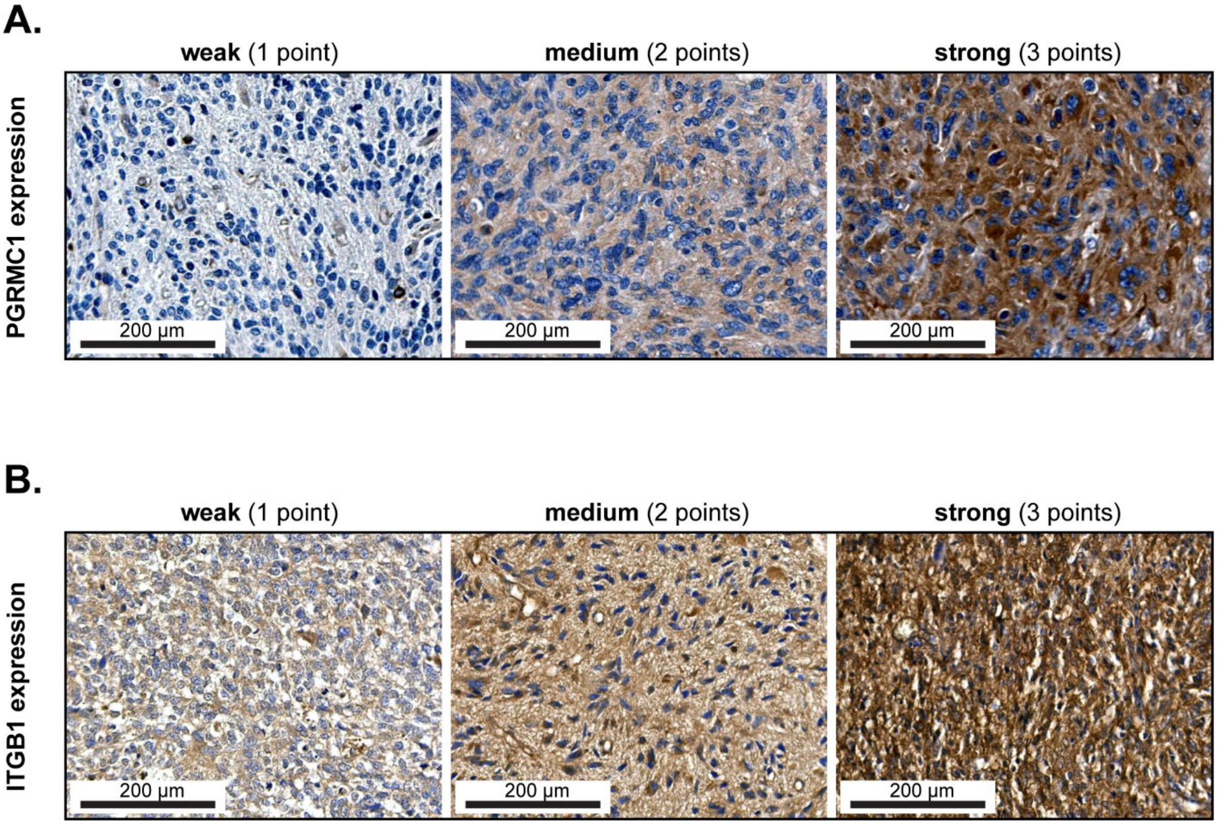

Supplementary Figure S1. PGRMC1 and ITGB1 scoring in GBM tissues. Representative micrographs showing weak (1 point), medium (2 points) and strong (3 points) expression of **(A)** PGRMC1 and **(B)** ITGB1 in GBM tissues. The H-score was subsequently calculated according to the formula: (1 x X) + (2 x Y) + (3 x Z), where X + Y + Z = 100% of the total tumor area. The scale bars are indicated in the lower-left corner of each panel.

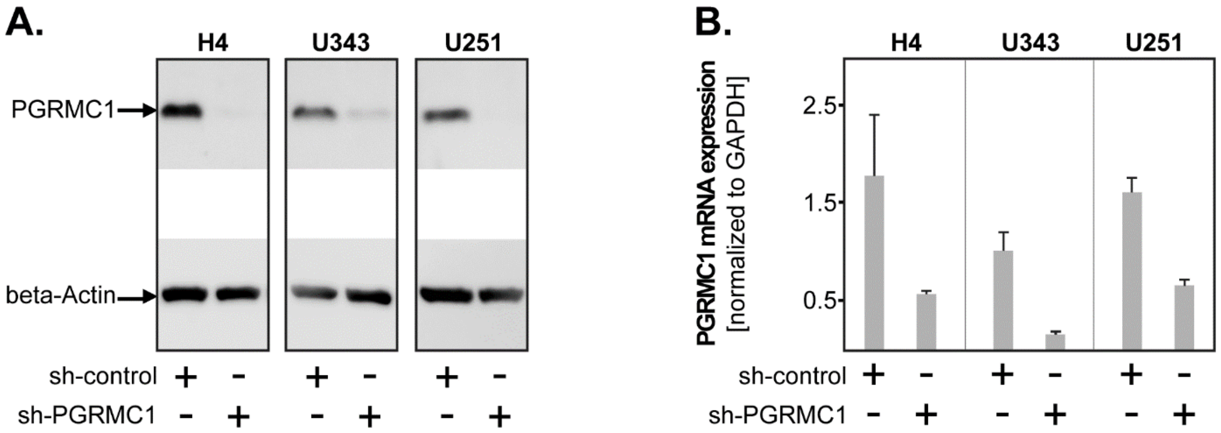

Supplementary Figure S2. Stable knockdown of PGRMC1 in GBM cell lines. H4, U343 and U251 cells were transfected with PGRMC1 shRNA (sh-PGRMC1) or a control shRNA (sh-control). **(A)** The expression of PGRMC1 at protein level was determined by western blot. Beta-Actin was used as loading control. **(B)** PGRMC1 mRNA levels in knockdown versus control cells. GAPDH was used as housekeeping gene to normalize the expression of PGRMC1 in these samples.

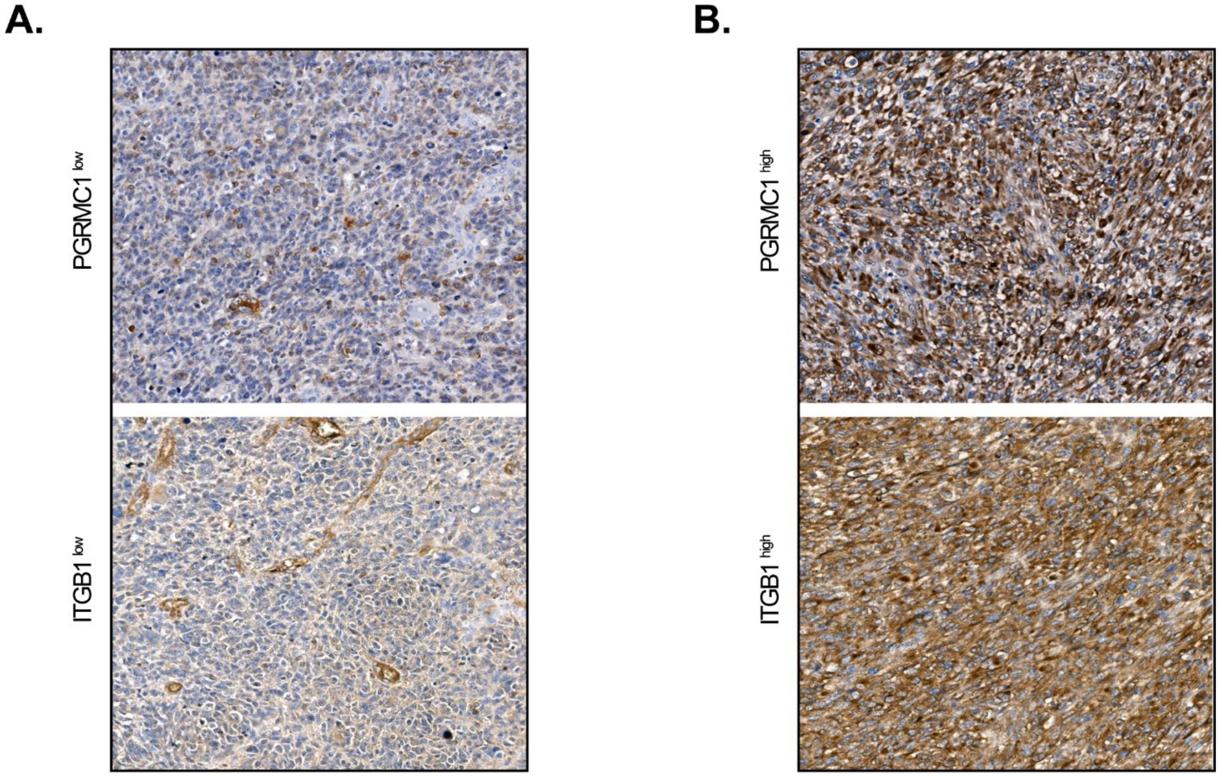

Supplementary Figure S3. PGRMC1 and ITGB1 synchronous expression in GBM tissues. Representative micrographs of GBM tissues with **(A)** synchronous low levels and **(B)** synchronous high levels of PGRMC1 and ITGB1.

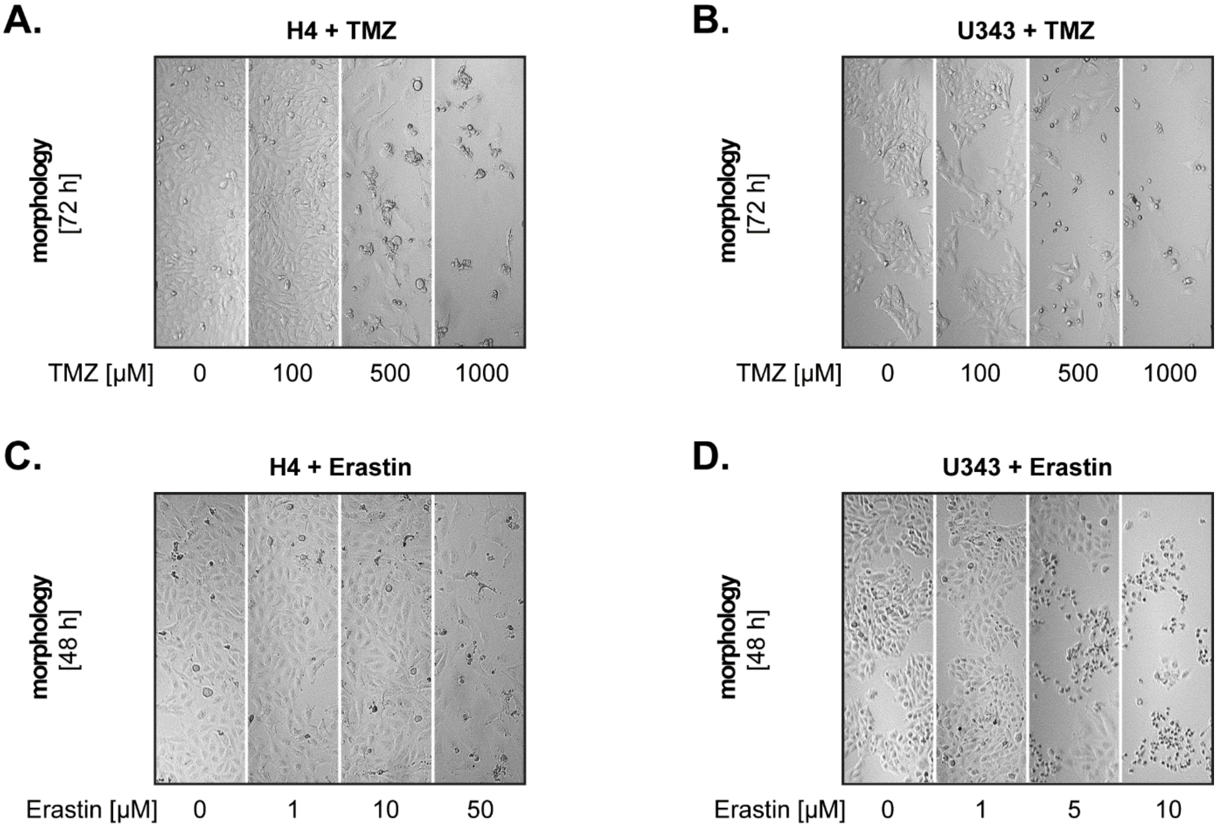

Supplementary Figure S4. TMZ and Erastin titration curves in GBM cell lines. Representative phase-contrast micrographs of **(A)** H4 and **(B)** U343 wild-type cells exposed to different doses of TMZ for 72 h. Representative phase-contrast micrographs of **(C)** H4 and **(D)** U343 wild-type cells exposed to different doses of Erastin for 48 h.

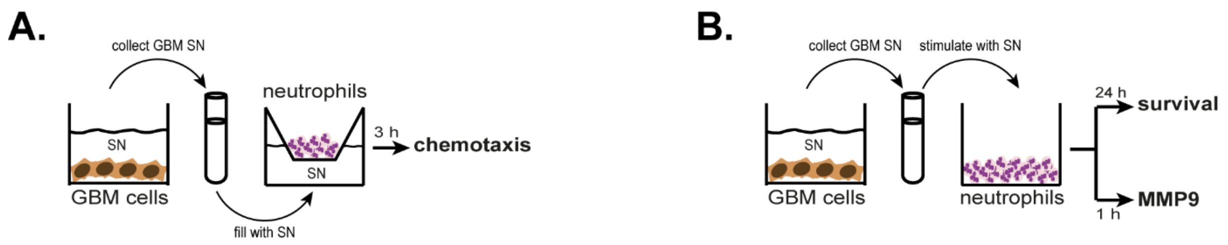

Supplementary Figure S5. Experimental design to investigate the GBM-neutrophil interactions *in vitro*. GBM cells were incubated for 24 h in culture medium to produce conditioned supernatants (SN). **(A)** The GBM SN were added to the lower and the neutrophils (re-suspended in culture medium) to the upper compartment of a transwell system. The number of neutrophils which migrated into the lower compartment was determined after 3 h incubation at 37°C. **(B)** Neutrophils were stimulated directly with GBM SN. Neutrophil survival was determined by Annexin V/Propidium Iodide staining after 24 h and the release of MMP9 by gelatin zymography after 1h in culture.
